# A matter of availability: sharper tuning for memorized than for perceived stimulus features

**DOI:** 10.1093/cercor/bhad064

**Published:** 2023-03-31

**Authors:** Samson Chota, Surya Gayet, J Leon Kenemans, Christian N L Olivers, Stefan Van der Stigchel

**Affiliations:** Experimental Psychology, Helmholtz Institute, Utrecht University, Heidelberglaan 8, Utrecht, the Netherlands; Experimental Psychology, Helmholtz Institute, Utrecht University, Heidelberglaan 8, Utrecht, the Netherlands; Experimental Psychology, Helmholtz Institute, Utrecht University, Heidelberglaan 8, Utrecht, the Netherlands; Department of Experimental and Applied Psychology, Vrije Universiteit Amsterdam, Van der Boechorststraat 1, 1081 BT Amsterdam, The Netherlands; Experimental Psychology, Helmholtz Institute, Utrecht University, Heidelberglaan 8, Utrecht, the Netherlands

**Keywords:** working memory, perception, EEG multivariate pattern analysis, embodied cognition

## Abstract

Our visual environment is relatively stable over time. An optimized visual system could capitalize on this by devoting less representational resources to objects that are physically present. The vividness of subjective experience, however, suggests that externally available (perceived) information is more strongly represented in neural signals than memorized information. To distinguish between these opposing predictions, we use EEG multivariate pattern analysis to quantify the representational strength of task-relevant features in anticipation of a change-detection task. Perceptual availability was manipulated between experimental blocks by either keeping the stimulus available on the screen during a 2-s delay period (perception) or removing it shortly after its initial presentation (memory). We find that task-relevant (attended) memorized features are more strongly represented than irrelevant (unattended) features. More importantly, we find that task-relevant features evoke significantly weaker representations when they are perceptually available compared with when they are unavailable. These findings demonstrate that, contrary to what subjective experience suggests, vividly perceived stimuli elicit weaker neural representations (in terms of detectable multivariate information) than the same stimuli maintained in visual working memory. We hypothesize that an efficient visual system spends little of its limited resources on the internal representation of information that is externally available anyway.

## Introduction

The brain is the most energy-demanding organ in the human body, accounting for ~20% of total energy consumption (Magistretti and Allaman 2015). Important cognitive processes implemented by neural activity are metabolically costly and therefore need to be highly optimized. One of these crucial processes is working memory, often defined as a short-term storage for information that is no longer available (Baddeley 1992). Working memory is surprisingly limited, with the average person only being able to remember around 3–5 objects depending on their complexity and the task context (Cowan 2010). This limitation is often explained by a resource bottleneck, further emphasizing the need for efficient distribution of resources in the brain (Cowan 2010). Various lines of research have indeed suggested that storing information in working memory is an energy-demanding process. For instance, poor performers in a working memory task show higher metabolic expenditure as compared with good performers, an effect that was specific for memory load (Backs and Seljos 1994). Furthermore, the pupil, commonly interpreted as a marker of cognitive load or effort, shows greater dilation with increasing working memory load as well as perceptual load (Kahneman and Beatty 1966; Just et al. 2003; Alnæs et al. 2014; Castaldi et al. 2021). Given that representing information incurs such costs in terms of capacity and effort, the visual system potentially developed sophisticated strategies to decide when active maintenance of such representations is necessary and when not.

Interestingly, we find that in more ecological contexts, e.g. when giving participants the choice on how many items to encode in WM at a time, very little information is actually maintained internally (Ballard et al. 1995; Somai et al. 2020; Draschkow et al. 2021; Sahakian et al. 2023). These findings suggest that the brain uses its limited representational resources sparsely, supporting an energy-efficient theory of working memory (Somai et al. 2020; Van der Stigchel 2020). This account predicts that only the minimal amount of information is stored to the largest effect. Importantly, however, this minimal amount of information that we decide to store internally in order to perform a certain task was shown to be dependent on several environmental factors such as locomotive demands (Draschkow et al. 2021) and time (Somai et al. 2020; Sahakian et al. 2023). When large head movements are required between the encoding and retrieval of single objects in working memory, participants tend to encode more items in between movements, thus optimally balancing the relative energy costs of movement and storage (Draschkow et al. 2021; Sahakian et al. 2023).

Our work falls into a larger movement, suggesting to study internal representations in the context of embodied cognition (O’Regan 1992; Ballard et al. 1995; Van der Stigchel 2020). These theories highlight the need to study mental processes, particularly working memory, in their interaction with an energy-dependent physical body and its environment. Such theories lead to a clear prediction: fewer resources should be allocated to the internal representation of stimuli that stay physically available in the outside world as compared with stimuli for which perceptual availability is temporally limited. Crucially, at the neural level, this leads to the counterintuitive prediction that vividly perceived stimuli should be represented less strongly than invisible but remembered stimuli. Here, we aimed to directly test this prediction by investigating how much information, as revealed by multivariate pattern analysis in the electroencephalography (EEG), is stored internally when visual information remains physically available in the external world.

Traditionally, visual perception has often been characterized as nearly unlimited in its capacity, an intuition that is doubtlessly influenced by our incredibly rich subjective experience of the world. Representing information necessarily costs resources, however, and converging evidence suggests that the brain’s capacity to represent currently available and no longer available information is equally limited, potentially due to a shared attentional resource (Tsubomi et al. 2013; Lapierre et al. 2017). Specifically, Tsubomi and colleagues presented participants with a classical change detection task in which several colored objects were either presented shortly (memory/absent condition) or for an extended period of time (perception/present condition). Importantly, in the present condition, there was no empty delay interval between sample and probe, and hence, the relevant visual information stayed continuously physically available in the outside world. Surprisingly, the authors reported that performance in both conditions was identical and that the contralateral delay activity (CDA), a common electrophysiological marker of working memory capacity, was indistinguishable between conditions. They conclude that representational capacity limitations are similar for information that stays perceptually available as compared with information that is only briefly available. On the one hand, this shows that efficient resource management seems equally necessary when information stays perceptually available, supporting the idea that physically available stimuli should be represented less strongly. Simultaneously, however, the presence of the CDA could be interpreted as evidence that perceptually available stimuli were represented equally strong in both conditions, counter to the idea that availability should modulate representational strength. It is currently not clear if the CDA directly reflects the actual storage of information in working memory or if it reflects an attentional process that can also be found, e.g. during multi-object tracking (Drew and Vogel 2008; Drew et al. 2012, 2013). These limitations—that are inherent to univariate response measures—motivated our decision to use multivariate pattern analysis (MVPA) to quantify the multivariate evidence of internal representations when stimuli were perceived versus when they were memorized. Notably, visual perception and visual working memory have been hypothesized to rely on overlapping populations, a claim usually referred to as the sensory recruitment hypothesis (Ester et al. 2009, 2016; Serences et al. 2009). This view also finds support in the cross-generalization of neural patterns during perception and during memory maintenance of the same stimuli (e.g. Harrison and Tong 2009; Rademaker et al. 2019). Stimulus-specific information could therefore be expected to evoke similar patterns of neural activity, irrespective of whether the stimulus is physically present or not. Here, we test this hypothesis by quantifying the multivariate evidence for identical stimulus features in the EEG signal, both during perception and memory.

In addition to perceptual availability, we manipulated feature relevance in our task. This served as a baseline against which we could compare the effects of availability, as relevant information should be represented more strongly than irrelevant information (both for physically present and absent stimuli). The effect of feature relevance on working memory representations has been investigated in the past: previous work found stronger representations for task relevant as compared with task irrelevant features (EEG: Bocincova and Johnson 2019; functional magnetic resonance imaging (fMRI): Serences et al. 2009; Yu and Shim 2017). Here, we were interested if the same holds true if object features stay perceptually available for a prolonged period.

To summarize, the current study aims to reveal how perceptual availability influences the strength with which features are represented in the brain; specifically, asking whether representations of perceptually available features will be represented more strongly (in line with their more vivid subjective experience) or less strongly (in line with a resource-efficiency stance). We investigate this in a blocked 2 by 2 experimental designs in which participants were presented with colored gratings. These gratings were either presented for 150 ms (absent condition) and hence had to be remembered or remained available on the screen for an extended period of time (1,850 ms). In half of the trials, participants were asked to compare the gratings’ color (attend color condition) to a subsequent probe, in the other half participants had to compare the spatial frequency (attend spatial frequency condition). We used time-resolved multivariate pattern analysis to decode the spatial frequency of the stimuli in all 4 conditions.

Our findings show that task relevance and stimulus availability jointly modulate representational strength of visual information. Specifically, we found that (i) perceptual availability decreased the amount of multivariate evidence for a feature regardless of its relevance for the task at hand, whereas (ii) task-relevance increased the amount of multivariate evidence only for features that were not perceptually available (i.e. maintained in memory). These findings are particularly remarkable as stimulus features in the present condition were directly attended and perceived and hence appeared far more vivid as compared with when the same feature was absent and thus maintained in working memory. We conclude that the visual system efficiently evaluates how much resources to dedicate to the representation of stimuli, and that factors, such as perceptual availability and relevance, bias this evaluation.

## Materials and methods

### Participants

A total of 26 participants (aged 21–30, 16 females) with normal or corrected to normal vision enrolled in the experiment. None of the participants reported a history of psychiatric diagnosis. Informed consent forms were signed before the experiment. The study was carried out in accordance with the protocol approved by the Ethics Committee of the Faculty of Social and Behavioral Sciences of Utrecht University and followed the Code of Ethics of the World Medical Association (Declaration of Helsinki). Subjects were compensated with 10 Euro/h.

### Stimuli

Stimuli consisted of vertical gratings (diameter 10° dva) with spatial frequency randomly selected from a set of 48 frequencies on every trial [1 cpdva (cycles per degree visual angle) to 4 cpdva, equally spaced] and were presented at fixation. Stimulus color was selected randomly from a set of 48 colors drawn from a circle in CIELAB color space (*L* = 54, *a* = 18, *b* = −8, radius = 59). A circular patch around the fixation point with radius 0.5° dva (degree visual angle) was cut out and the inner edges of the resulting annulus were blurred in order to prevent participants from basing their spatial frequency judgment on the relative position of grating and fixation cross. Similarly, we blurred the outer edges of the gratings since spatial frequency judgments might be based on the patterns of these sharp transition zones. Stimuli were presented on an LCD display (27-inch, 2560 × 1440 resolution, 120-Hz refresh rate) using the Psychophysics Toolbox running in MATLAB (MathWorks). Participants were seated 58 cm away from the screen on a chinrest to prevent excessive head movements.

### Protocol

**Fig. 1:**
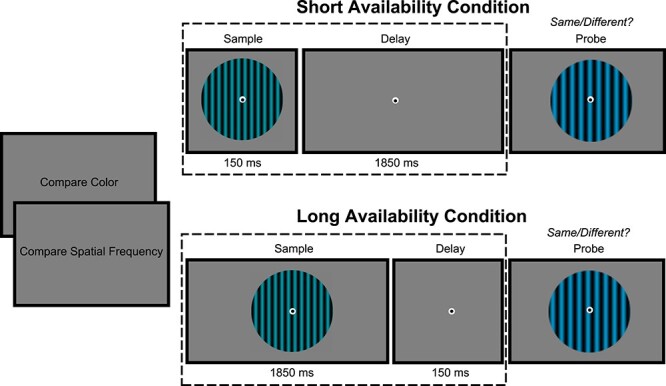
Experimental paradigm. Participants performed a delayed match-to-sample task. In “compare spatial frequency” blocks, participants reported if the spatial frequency of the probe differed from the sample or not. Similarly, in “compare color” blocks, participants compared the color of the probe to that of the sample. The duration for which the sample was presented on the screen also varied in a blocked fashion, leading to 4 different types of blocks (compare SF short availability, compare SF present, compare color short availability, compare color present).

Participants performed a blocked delayed match-to-sample task (Fig. 1). Participants were instructed to remember the spatial frequency or the color of vertically oriented gratings and compare them to the respective feature of the subsequent probe. At the beginning of each block, participants were informed about the relevant feature in this particular block (“Attend Color” versus “Attend Spatial Frequency”). Furthermore, participants were informed if the sample would stay on the screen or be removed during the delay interval (“Present Condition” versus “Absent Condition”). In the present condition, the sample was presented for a total duration of 1850 ms followed by an empty screen for 150 ms. In the absent condition, the sample was presented for 150 ms followed by an empty screen for 1,850 ms. The relevance and presence were kept constant for the entire block. Hence, participants had clear expectations on how long the stimuli would be available, and 500 ms after the onset of the probe the fixation cross turned green, instructing the participant to report if the relevant feature was the same or different (50% probability) as compared with the sample. Participants performed a total of 1,000 trials. The number of trials per condition was matched. To keep the task equally difficult between all conditions, we used an online staircase procedure (Psychtoolbox QUEST algorithm) that was updating several psychometric functions based on performance on every trial. The difference in spatial frequency and color between sample and probe was chosen based on these dynamic psychometric functions so that performance was approximately at 75% in all conditions (1. Attend color short availability, 2. Attend color long availability, 3. Attend SF short availability and 4. Attend SF long availability). Within every condition, 5 psychometric functions were used, 1 for color and 4 for the 4 bins of spatial frequencies (1–1.75, 1.76–2.55, 2.56–3.25, 3.26–4 cpdva). We used several psychometric functions for spatial frequency bins because the “just noticeable difference” likely varies across different SF. At the end of every block, participant received feedback in the form of a percent correct report. At the beginning of the experiment, participants performed a practice session comprising 5 trials of each condition in which immediate feedback was provided.

### Eye tracking recording and analysis

Gaze position was continuously tracked using an Eyelink 1000 (SR Researcher) eye tracker. Eye tracker calibration was performed at the beginning of the experiment and the gaze position was sampled at 1,000 Hz. Saccades were extracted directly from the EyeLink saccade detection algorithm. Per condition, we calculated the average number of saccades per trial as well as the average saccade amplitude.

For the Gaze position decoding analysis, we separated trials (vertical and horizontal position over time) into 4 different groups corresponding to the spatial frequencies used in the EEG decoding procedure. We performed baseline correction (−500 to 0 ms relative to stimulus onset) on the signal to remove slow drifts. Similar to the EEG decoding analysis, we divided the gaze trials into 3 equal groups of which 2 served for training and 1 served as test set (3-fold cross-validation). MATLAB’s *fitcecoc* function (SVM, one-versus-all) was used to fit linear classifiers on 2 of the folds and test it on the 3rd fold. Training and testing was performed on the same time point and was repeated 10 times, each time shuffling the trials that were divided into the 3-folds.

### E‌EG recording and preprocessing

We recorded participants EEG using a 64 channel ActiveTwo Biosemi system. Two additional electrodes placed on the outer eye canthus and above the left eye recorded horizontal and vertical eye movements. Data analysis was performed in MATLAB using the Fieldtrip toolbox. Prior to all preprocessing steps, we identified and removed bad channels via visual inspection. The EEG data were then re-referenced to the average of channel T7 and T8, bandpass filtered between 0.01 and 80 Hz, and line noise was removed using a DFT-filter (50 Hz). Thereafter, the data were epoched from 1.5 s before sample onset to 3.5 s after sample onset. Large muscle and head-movement-related artifacts were first removed through visual inspection. Afterward, we performed an independent component analysis (ICA) on the datasets separately to remove eye-movement-related artifacts. Finally, the data were down sampled to 100 Hz and absolute baseline correction was performed (window −500 to 0 ms before sample onset). We removed two subjects from the analysis due to a large number of EEG artifacts (>25% trials removed) caused by excessive head or eye movements during the experiment. All data is available on request.

### Multivariate pattern analysis

We decoded the spatial frequency of the sample stimulus based on the scalp topographies of the ERP voltage using a similar procedure as Bae and Luck (2018). We chose to train our decoders only on trials in which participants provided correct responses as these trials would reflect proper attention and memory allocation. The number of trials per spatial frequency class was equalized before training multivariate classifiers. Single trial ERPs were lowpass filtered using an infinite impulse response butterworth lowpass filter (cutoff frequency 8 Hz) and subsequently down sampled to 50 Hz. We decided to decode the spatial frequency from a selection of 20 occipital channels (PO7, PO3, O1, Oz, POz, Iz, PO8, PO4, O2, P1, P2, P3, P4, P5, P6, P7, P8, P9, P10, Pz) as we were mainly interested in visual representations.

For every participant, we binned trials into 4 spatial frequency classes of equal size. Trials in every class were divided into 3 equally sized groups and averaged, resulting in 3-folds that were later used for 3-fold cross-validation. We used MATLAB’s *fitcecoc* function to fit multiclass error correcting output code models using support vector machines that were trained to distinguish between 1 class and all others (one-versus-all). For every individual separately, classifiers were trained on single timepoints and received 2 samples from each of 4 SF classes consisting of 20 features in the form of EEG topography voltage (20 channels). Classifiers were then tested on all timepoints using the remaining fold (4 classes, 20 features). This procedure was repeated 3 times with each fold serving as training and test set once, but never as both simultaneously. In addition, we randomly shuffled the trials that were averaged into each fold 10 times, each time repeating the entire decoding procedure. The classifiers output scores (distance-to-bound) were z-scored for normalization, multiplied with the true class labels (1 or −1), and then averaged for individual timepoints.

### Statistical analysis

We statistically assessed the diagonal distance-to-bound decoding scores using a nonparametric cluster-based permutation test (Maris and Oostenveld 2007). This was done first to assess if significant above chance decoding could be observed within conditions and second, using a different approach, to test if decoding significantly differed between conditions. To statistically assess decoding within a condition, we first simulated the performance of our decoder that would be observed when guessing randomly. Notably, all the following analyses were performed on the de-signed distance-to-bound scores (positive for correct and negative for incorrect classification). For every participant and trial, we randomly generated either a correct or a false classification value by multiplying the entire distance-to-bound scores time-series with 1 or −1 (thus preserving autocorrelations between time-points in the null data). Distance-to-bound time-series were averaged across trials within participants. We then ran *t*-tests for every individual timepoint of the group level decoding time-series and identified clusters of consecutive timepoints where the *P*-value fell below α = 0.05. The *t*-values within the largest cluster were summed to calculate the cluster-level *t* mass. This procedure was repeated 10.000 times to generate a distribution of cluster-level *t* masses. We then compared the experimentally observed cluster-level *t* masses to the null-distribution and rejected the null hypothesis (H0: distance-to-bound score is not significantly different from 0) if their mass exceeded the 95% quantile of the null-distribution. We performed an identical procedure for the decoding based on gaze data.

In order to compare decoding scores between 2 experimental conditions, we performed a similar statistical permutation procedure. First, we calculated the veridical difference in cluster-level *t* mass between 2 conditions. We then randomly swapped 50% of labels in both conditions and recalculated the difference in largest cluster-level *t* mass. This procedure was repeated 10.000 times and generated the distribution that would be expected under the null hypothesis (H0: distribution of distance-to-bound score *t*-mass differences do not differ significantly between conditions). The observed *t*-mass difference was compared with the null-distribution and the null hypothesis was rejected if it exceeded the 95% quantile.

Notably, the results of cluster-based permutation tests do not indicate which time-points cause the observed difference between conditions, but only allow to reject the null hypothesis (H:0 both conditions come from the same probability distribution) (Maris and Oostenveld 2007).

Behavioral performance (accuracy) was analyzed using a 1-way ANOVA with factors availability and relevance.

## Results

### Behavior

**Fig. 2:**
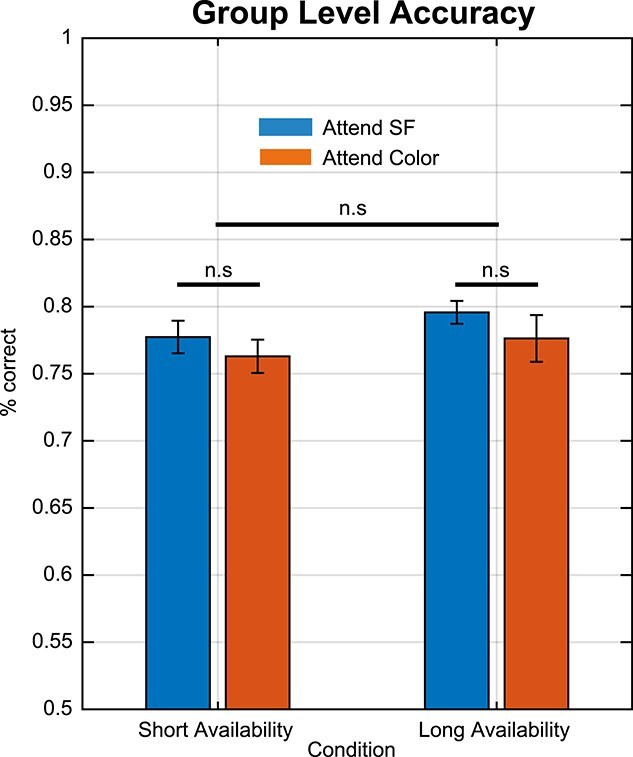
Behavioral accuracy. Group average performance for all 4 conditions. Error bars indicate standard error of mean.

A 2-way ANOVA with factors relevance (SF vs Color) and availability (short availability vs long availability) revealed no main effect of relevance (*F*(1,108) = 1.683, *P* = 0.197) or availability (*F*(1,108) = 1.483, *P* = 0.226) and no interaction effect between the two (*F*(1,108) = 0.037, *P* = 0.847), indicating that our staircase procedure successfully equalized task difficulty between conditions (Fig. 2). The average staircase values for SF, indicating the difference in SF between memory and probe, were significantly smaller in long vs short availability conditions (1–1.75 cpdva: *P* < 0.05; 1.76–2.55 cpdva: *P* < 0.05; 2.56–3.25 cpdva: *P* < 0.05; 3.26–4 cpdva: *P* < 0.05; 2-tailed *t*-test). Similarly, the average staircase values for color were significantly smaller in the long availability condition (*P* < 0.05 2-tailed *t*-test).

### Effect of availability and relevance

We trained linear classifiers to quantify the time-resolved separability of the EEG signal, acquired during and after the presentation of visual gratings with varying spatial frequencies and color. Stimuli were presented either for 150 ms followed by a blank delay interval of 1850 ms (short availability condition) or remained on the screen for 1850 ms, followed by a blank delay interval of 150 ms. In the compare spatial frequency condition, participants had to indicate if the probe had a similar or different SF as compared with the sample. In the color condition, participants indicated if the stimulus color was the same or different between sample and probe.

**Fig. 3:**
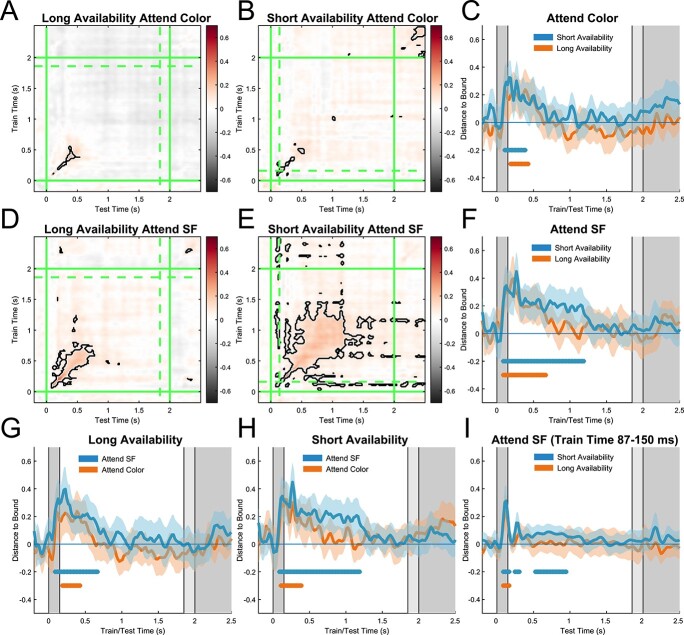
EEG decoding. A) Temporal generalization matrix reflecting classifier performance for long availability attend SF condition. *Y*-axis indicates timepoints on which classifiers were trained. *X*-axis indicates timepoints on which classifiers were tested. Color axis indicates mean normalized distance-to-bound scores. Green lines indicate onset (solid) and offset (dashed) of stimuli in the respective condition. B) Same but for short availability attend color, D) long availability attend SF, and E) short availability attend SF conditions. C) Diagonal decoding performance (timepoint-by-timepoint) of short availability (blue line) and long availability (orange line) attend color conditions. Colored areas indicate 95% confidence intervals. Colored bars (blue, orange) indicate significant clusters of above chance decoding. Gray bars in the background indicate onset and offset of stimulus in short availability and long availability conditions respectively. F) Same but for attend SF short availability (blue) and long availability (orange) conditions, G) long availability attend SF (blue) and attend color (orange) conditions, H) short availability attend SF (blue), and attend color (orange) conditions. I) Short availability attend SF (blue) and long availability attend SF (orange) conditions. Classifiers were trained on early timepoints (87–150 ms) of pooled availability conditions (attend SF) and were tested on all timepoints for each condition separately.

Visual inspection of the MVPA temporal generalization matrices, displaying decoding performance (distance-to-bound) for all combinations of training and testing timepoints, indicated that both relevance and availability modulated decodability (Fig. 3A, B, D, E). Statistical analysis of the timepoint-by-timepoint decodability in the latency range from 0 to 2,000 ms after stimulus onset revealed a significant difference between conditions in which SF was the relevant feature as compared with when it was the irrelevant feature (Fig. 3G: short availability relevant vs short availability irrelevant, cluster-based permutation test: *P* < 0.0001, Fig. 3H: long availability relevant vs long availability irrelevant, cluster-based permutation test: *P* < 0.0005). Decoding time-series indicated that this was because SF could be decoded longer in SF relevant (short: 1,180 ms, long: 660 ms) as compared with SF irrelevant conditions (short: 380 ms, long: 420 ms).

Most importantly, we found significant differences between the short availability and the long availability condition when SF was relevant (Fig. 3F, cluster-based permutation test: *P* < 0.0125), most likely due to prolonged decoding in the short availability condition (short: 1,180 ms, long: 660 ms). This result is particularly striking because participants were directly fixating and perceiving the grating in the long availability condition for almost 2 s and were aware that the spatial frequency was relevant for successful probe comparison. There is a possibility that the difference observed between these conditions is caused by the offset response of the stimulus, which only occurred during the analysis time-window of the short availability condition. When SF was not relevant, however, no significant difference was found between short and long availability conditions (cluster-based permutation test: *P* > 0.1875, Fig. 3C). Thus, the offset response did not prolong decodability when SF was irrelevant. Accordingly, it is unlikely that the larger decoding cluster found in the short availability SF relevant condition was solely caused by the offset of the sample stimulus at time 150 ms.

To test whether the differences in multivariate evidence between long and short availability conditions are caused by a difference in the training set rather than differences in the actual amount of information that is represented during the testing timepoints, we conducted an additional analysis keeping the training set stable. All trials began with the presentation of a colored grating and long and short availability conditions only differed in the timepoint at which this grating disappeared (150 vs 1850 ms). Throughout the first 150 ms, all trials should therefore be comparable between availability conditions and classifiers should be able to capture (and generalize) the sensory signals evoked by the stimulus onset (Harrison and Tong 2009; Rademaker et al. 2019). This allowed us to pool both conditions and train classifiers on the early timepoints of a single, instead of 2 different training sets. The classification analysis was kept identical to the within condition analysis, with the exception that classifiers were trained on both conditions (attend SF, long and short availability) and only on the timepoints from the classification onset (Fig. 3F, 87 ms) to the disappearance of the stimulus in the short availability condition (150 ms). Subsequently, classifiers were tested on all timepoints, separately for short and long availability conditions, to quantify how well multivariate early sensory representations generalize to long and short availability conditions. We only investigated the attend SF condition as it was most relevant for our hypothesis. In line with our previous findings, we found significant differences between the short and long availability condition when SF was relevant (Fig. 3I, cluster-based permutation test: *P* < 0.005), most likely due to prolonged decoding in the short availability condition. This result suggests that the differences in multivariate evidence between availability conditions are not caused by differences in the training set and/or fundamental differences in the neural codes employed during perception and memory.

Given these results we conclude that the lack of sustained decoding in the *long availability* relevant condition might be a result of the long availability of the stimulus. As participants were aware that the relevant feature will be perceptually available in the external world for at least 1,850 ms, the visual system likely has no incentive to engage large amounts of resources to represent the feature. Alternatively, participants might have not attended the stimulus in the “long availability” condition as much as the only shortly available stimulus in the long availability condition, leading to differences in decoding performance. Our following analysis aimed to address this possibility.

### Attention decoding

To test whether participants dedicated few or none of their attentional resources to the long availability stimulus at its onset, we conducted an additional decoding analysis. Our rationale was as follows: if participants did not attend the stimulus in the long availability condition because they were aware of its continuous presence then the neural signals in long availability relevant and long availability irrelevant conditions should indistinguishable. Conversely, if participants attended the long availability stimulus, then a classifier should be able to distinguish between long availability relevant and irrelevant conditions.

**Fig. 4:**
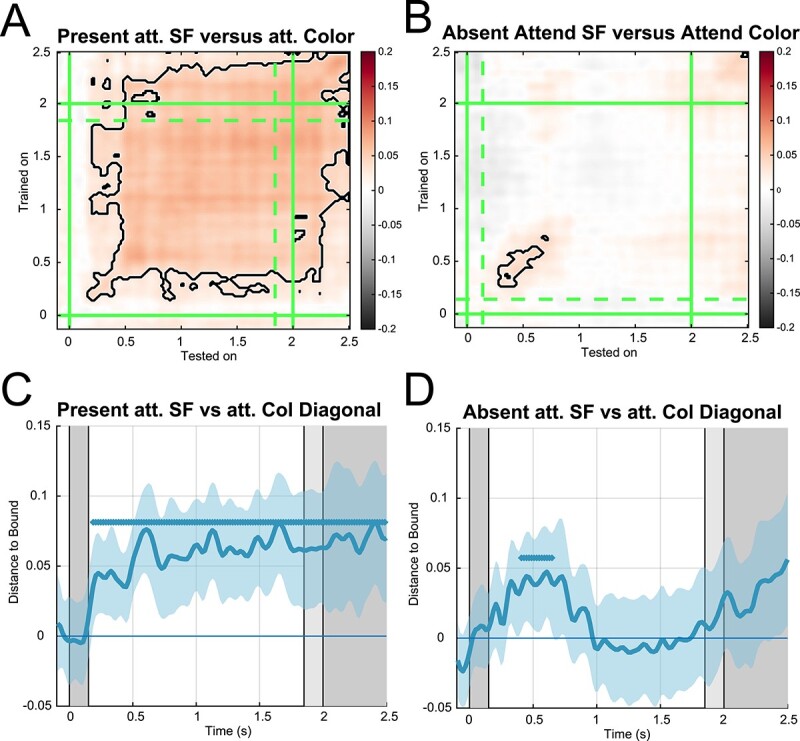
Attention EEG decoding. A) Temporal generalization decodability matrix for SF relevant versus irrelevant. Color axis indicates mean normalized distance-to-bound scores. Green lines indicate onset (solid) and offset (dashed) of stimuli in the respective condition. B) Same but for short availability condition. C, D) Diagonal decoding (timepoint-by-timepoint) of and long availability and short availability conditions. Colored areas indicate 95% confidence intervals. Blue colored bars indicate significant clusters of above chance decoding.

We trained a new set of binary support vector machines to distinguish between trials from the long availability relevant and irrelevant conditions, regardless of their spatial frequencies. Classifiers successfully distinguished between long availability relevance conditions as early as 187 ms after stimulus onset indicating that attentional signals differed between them (Fig. 4A, C, *t*-tests). This finding, together with the fact that we found a significant effect of relevance in the long availability condition, suggests that the poor SF decoding found in the long availability relevant condition was likely not caused by a lack of attention at stimulus onset nor during the rest of the delay.

### Classification of SF from gaze

**Fig. 5:**
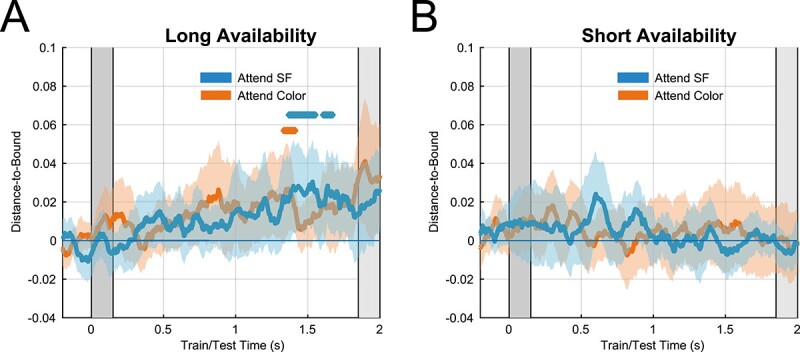
Decoding spatial frequency from eye positions. Timepoint-by-timepoint decoding of SF from gaze positions. A) Long availability condition SF relevant (blue) and irrelevant (orange). Colored areas indicate 95% confidence intervals. Gray bars in the background indicate onset and offset of stimulus in short availability and long availability conditions, respectively. Colored bars (blue: SF relevant, orange: SF irrelevant) indicate significant clusters of above chance decoding. B) Same but for short availability condition.

Eye movements were previously shown to systematically track orientation features held in working memory and can lead to decodable artifacts in the EEG signal (Mostert et al. 2018). We considered this possibility in the design of our task and choose to use spatial frequency instead of spatial orientation as decodable feature, as this would not provide spatial locations, such as orientation endpoints to bias eye movements that were correlated with our decoding variables. To test if this was successful, we attempted to decode the SF from gaze position alone using a similar decoding analysis as was used for the EEG decoding. Classifiers could only briefly distinguish between different SF based on gaze location (horizontal, vertical position) in the long availability condition and only after 1,400 ms (Fig. 5). Notably, this time-period does not overlap with the time-period of significant EEG decoding (0–1180 ms). Our EEG decoding results are therefore unlikely to be a result of eye-artifacts caused by systematic eye movements.

## Discussion

Here, we investigated whether continuously physically present stimuli lead to less distinguishable neural patterns as compared with memorized stimuli using EEG multivariate pattern analysis (MVPA). Our first hypothesis was that classifiers would perform worse when stimuli remained visible as the visual system could potentially externalize their storage to the physical world. This was tested by changing the duration for which stimuli would remain available on the screen in a predictable fashion. We were particularly interested in the manipulation of perceptual availability because subjective experience and an efficient theory of neural representations make opposite predictions on how availability should affect stimulus-specific activity. Using MVPA, we found that stimulus features that remained available for a long period were accompanied by significantly worse classifier performance as compared with only shortly available features, although the latter are commonly subjectively experienced as far less vivid. This was also the case when classifiers were trained only on the initial sensory transients which generalized to later periods in a trial. We theorize that the reduction in classifier performance is caused by a reduction in stimulus-specific activity, as the visual system has no incentive to represent readily available visual information. We also hypothesized that higher feature relevance, an additional factor orthogonal to perceptual availability, should lead to better classification accuracy. Our findings confirmed this prediction, further supporting the idea that encoding and maintenance of neural representations is influenced by various task demands (Ballard et al. 1995; Alfandari et al. 2019; Somai et al. 2020; Van der Stigchel 2020; Draschkow et al. 2021; Sahakian et al. 2023).

The effects of relevance (within the long availability condition) are in line with previous accounts of feature-based attention during continued perception that generally show better decoding for relevant as compared with irrelevant features, when stimuli are perceptually available (Kamitani and Tong 2005, 2006; Moerel et al. 2022). Importantly, the stimuli in these studies were either flickering on and off (Kamitani and Tong 2005; Moerel et al. 2022) or consisted of moving stimuli that continuously generated visual transients (Kamitani and Tong 2006). These design decisions were likely made to increase the ability to decode the attended features by maximizing the bottom-up sensory signal. Here, we show that the initial sensory multivariate response is indeed modulated by attention but additionally demonstrate that once the sensory transient disappears, multivariate pattern classification falls of sharply even though subjective visual perception remains unchanged.

An additional MVPA analysis aimed at dissociating between attentional conditions revealed that neural signals differed significantly between trials where attention was directed toward the spatial frequency and the stimulus color. This effect remained significant while the stimulus was displayed in the long availability condition but was short lived in the short availability condition. Importantly, this result shows that the perceived stimulus (long availability condition) was indeed attended throughout its presence and that the reduced classifier performance was not caused by a lack of attention to the presented stimulus.

Can the effect of availability on decoding accuracy be explained in terms of visual adaptation, a commonly observed reduction in visually evoked activity over time (Webster 2015; Benda 2021)? Visual adaptation has been observed on multiple time-scales in the visual system and can occur in time ranges matching the average duration of a fixation (~400 ms, Akyuz et al. 2020; Gutnisky and Dragoi 2008). Adaptation is highly prevalent in the brain, leading to several proposed fundamental functions such as efficient coding (Clifford et al. 2007; Wark et al. 2007), error correction (Andrews 1967), and predictive coding (Chopin and Mamassian 2012). While there are many EEG studies investigating long-term visual adaptation, we only found a single study that decoded visual stimuli that were statically perceived for an extended duration and that could therefore give insights on how adaptation changes decoding performance over time (500 ms, Carlson et al. 2011). Interestingly, although stimuli were relevant on every trial, decoding accuracy sharply dropped off after around 500 ms approaching chance, similar to what we found in our long availability condition. Although visual adaptation during continuous visual perception likely plays a role in explaining the reduced classification accuracy found in our and other studies (Carlson et al. 2011), several questions still remain open. First and foremost, visual adaptation does not explain the most puzzling of our results: the fact that the common qualitative vividness of visual experience is not accompanied by strong classification performance for the stimulus feature. Second, adaptation does not seem to occur in the short availability condition. At first glance, it might seem obvious that adaptation occurs to a lesser extent when there is no available sensory stimulus to adapt to. A priori however, there is no reason to assume that adaptation should not occur for neurons that are active for prolonged periods during the delay period without sensory input, especially because sensory populations seem to be shared between perception and working memory (Harrison and Tong 2009; Rademaker et al. 2019, our results Fig. 3I). From another perspective, adaptation serves a number of proposed functions including efficient coding (Clifford et al. 2007; Wark et al. 2007), error correction (Andrews 1967), and predictive coding (Chopin and Mamassian 2012). Assuming that working memory maintenance does not induce adaptation, implicitly assumes that these functional properties of adaptation do not play a role in working memory maintenance. This notion is in contrast with several experimental findings that propose a potential role of adaptation during short-term memory processes (Marder et al. 1996; Turrigiano et al. 1996), most recently in the context of language processing (Fitz et al. 2020). As the previously mentioned principles (efficient coding, error correction, and predictive coding) are hypothesized to be fundamental principles of neural coding, we consider it very likely that they are also involved in higher level cognitive functions such as working memory. Moreover, efficient coding is assumed to serve the reduction of metabolic cost by reducing stimulus-related spiking activity, for instance when sensory information stays unchanged (or available), which is consistent with our hypothesis (Clifford et al. 2007; Wark et al. 2007).

Although our MVPA analysis revealed little evidence for the spatial frequency of the stimuli after a few hundred milliseconds (short availability: 1180 ms, long availability 660 ms), it is unlikely that the information completely vanished from the brain and more likely reflects the limitations of our measurement instruments. Considering that participants were able to perform the task with reasonable accuracy the information must have been somehow stored but escaped our classifiers. This could be due to several reasons. First, it is plausible that the quality of our signal deteriorated over time due to slow drifts in the EEG signal (van Driel et al. 2021). Moreover, it has been proposed that neural representations can be stored in so called “activity-silent” states that rely on short-term synaptic changes to encode information (Mongillo et al. 2008; Chota and Van der Stigchel 2021). This latter possibility has mostly been discussed in the context of working memory; hence, it is not clear if this also applies to the representation of perceptual information.

Activity silent states are only one of several potential mechanisms supporting the representation of visual information during perception and working memory. Although work supporting the sensory recruitment hypothesis (Ester et al. 2009, 2016; Serences et al. 2009, results from our cross-generalization analysis Fig. 3I) suggests a certain degree of similarity, differences in how visual information is encoded in neural patterns could make certain patterns appear more similar when using a multivariate approach such as the one employed here. This could give the false impression that lower decoding accuracy in one condition is necessarily the result of less information in the neural signal instead of an increase in pattern similarity between stimulus classes. Although not being able to fully exclude this possibility, we ensured that at least functionally, similar representational systems had to be engaged between the long and short availability conditions, by keeping the stimulus sets, task, and difficulty as well as training sets for classification identical (Fig. 3I).

In a previous EEG decoding experiment, it was demonstrated that the relevant features of multi-object features could be decoded better than irrelevant features during the working memory delay period (Bocincova and Johnson 2019). Similar evidence was provided by fMRI studies showing significantly better decoding accuracy for relevant versus irrelevant features of items maintained in WM (Serences et al. 2009; Yu and Shim 2017). Our findings are in line with these results, showing that classification performance is decreased for irrelevant features during the delay period. Interestingly, several behavioral studies suggested that objects are stored in working memory as integrated features, the so-called object-based storage account (Luck and Vogel 1997; Vogel et al. 2001; Olson and Jiang 2002; Xu 2002a, 2002b, 2006; Awh et al. 2007; Fougnie et al. 2013). Some of these studies even show that capacity limitations do not significantly differ when subjects have to memorize the orientation, the color or both features of an object (Luck and Vogel 1997). While the present results and other decoding studies show that decoding accuracy is reduced or even at chance for irrelevant features, it is possible that the neural signals for these features are simply not decodable to the same extent as the relevant features, for instance because of lower signal to noise or differences in neural codes (van Loon et al. 2018; Chota and Van der Stigchel 2021; Iamshchinina et al. 2021). If the stimulus-specific delay activity for a certain feature is an indication of the effort that is required to maintain or keep the feature active (e.g. as search template), our current findings support the idea that irrelevant features are kept in a low effort state, e.g. a synaptic code (Mongillo et al. 2008).

Our findings are in support of an embodied account of working memory (Van der Stigchel 2020). Our visual environment and the physical objects it contains remain relatively stable over time. The brain can utilize this fact to optimize working memory usage by evaluating, e.g. which information will be lost if it is not currently encoded. This evaluation could be used to flexibly increase (due to high relevance or short availability) or decrease (due to irrelevance or long availability) the representational strength of object features. This important interaction between the external world, providing context and information, and working memory system has been largely ignored by traditional models of WM, who have investigated internal memory as isolated system, only engaged when information is no longer available. Our study provides an intriguing example of how these crucial factors can drastically alter multivariate classification—a likely proxy for representational strength—and why studying these interactions might be crucial for the study of WM.

We demonstrate that stimulus features that are memorized (but absent) can be significantly better classified using EEG MVPA compared with the same stimulus features when they are readily available and attended. Our findings highlight the role of two key factors, relevance and availability, that determine the quality of stimulus-specific visual representations. At the same time, our findings raise the intriguing question of why the quality of these visual representations in visual processing regions is not indicative of the vividness of subjective experience. Finally, our results speak to the embodied and economic use of limited representational resources in the brain.
